# Direct Transesterification for Biodiesel Production and Testing the Engine for Performance and Emissions Run on Biodiesel-Diesel-Nano Blends

**DOI:** 10.3390/nano11020417

**Published:** 2021-02-06

**Authors:** T. M. Yunus Khan

**Affiliations:** Department of Mechanical Engineering, College of Engineering, King Khalid University, Abha 61421, Asir, Saudi Arabia; yunus.tatagar@gmail.com

**Keywords:** Neem biodiesel, Karanja biodiesel, direct transesterification, graphene oxide nano particles

## Abstract

In the current research, the biodiesel was prepared from feedstocks of Neem oil and Karanja oil employing a single step direct transesterification method using acid-base catalysts simultaneously. The fuel properties of both Neem and Karanja biodiesel along with different biodiesel-diesel blends were studied and compared. Biodiesel produced from Neem oil was found better in terms of kinematic viscosity, calorific value and cloud point for all its blends with diesel compared to Karanja biodiesel-diesel blends. Experiments were conducted to study the effects of addition of graphene nano particles on fuel properties of biodiesel-diesel blends. The B20 biodiesel-diesel blend was selected, which was blended with graphene nano particles in different proportions (35, 70, 105 ppm) to get different stable and symmetric B20-nano blends. The fuel properties except kinematic viscosity were further improved with higher dosages of nano particles with the biodiesel-diesel blend. The performance and emissions tests were conducted on 4-stroke variable compression ratio diesel engine. Higher concentrated B20-nano blends of Neem (NOME20GO105) and Karanja (KOME20GO105) resulted in 31 and 30.9% of brake thermal efficiency, respectively, compared with diesel of 32.5%. The brake-specific fuel consumption (BSFC) was reduced by 10 and 11% for NOME20GO105 and KOME20GO105, respectively, compared to their respective B20 blends. Similarly, carbon monoxide (CO) was reduced significantly by 27 and 29% for NOME20GO105 and KOME20GO105, respectively.

## 1. Introduction

The transportation sector is a major contributor for poor air quality, acidification, carbon di oxide (CO_2_), and greenhouse gases [1]. These are the major concerns among the developing countries [2]. It is well known that transportation sector is contributing around 25% of the CO_2_ pollutant with the road transport sector alone is responsible for 80% [3]. Furthermore, it is anticipated that CO_2_ pollutant will further increase to 41% by the year 2030 compared to the data corresponding to 2007 [4]. The internal combustion (IC) engines are the backbone of transportation sector. Presently, 95% of the transport energy is obtained from combustion of fossil fuels. The demand for transportation energy is large around the globe and increasing too [5]. The other alternative sources of energy for transportation sector are biofuels, liquid petroleum gas and compressed natural gas, which constitute 5% of total transportation energy consumption. The contribution of electricity, synthetic fuels and hydrogen are negligible. There is a lot of interest shown by many researchers in the development and implementation of electric vehicles, fuel cell powered by hydrogen due to strict legislations regarding the emissions norms and deteriorating quality of air. However, there are still many challenges associated with their full-fledged implementation. Therefore, IC engines will continue to drive the transportation sector [6]. The excessive use of fossil fuels in IC engines has led to harmful effects on the environment and quality of living. These harmful effects could be minimized to a large extent by replacing the renewable fuels in compression ignition (CI) engines [7]. The legislatures and policy makers of European countries are recognizing the risks associated in using fossil fuels with regards to their harmful effects on the environment. European countries have been accelerating towards the development of renewable fuels for the transportation sector. Sweden has emerged successful among European countries in the process of greening energy [8]. Foteinis et al. [9] examined the environmental sustainability of used cooking oil (UCO) biodiesel and compared it with petro-diesel. The tests were conducted at industrial level in Greece. They reported a threefold reduction in environmental effects compared to petro-diesel. Furthermore, the authors suggested that UCO could play a key role in decarbonizing Europe’s transportation sector [9]. Biodiesel can be used in its pure form or blended with diesel in different proportions in an unmodified diesel engine. However, the viscosity and density of biodiesel-diesel blends are slightly more than that of diesel, which are the main obstacles for commercialization of biodiesel [10]. Ali et al. [11] concluded that biodiesel by-products must be fully utilized to make biodiesel a sustainable replacement of diesel [11].

Biodiesel may be commonly produced by using two-step esterification-transesterification methods [12]. However, the production methods for biodiesel have been undergoing through rapid scientific and innovative developments to commercialize it as an alternate fuel to the diesel. 

These developments are focused on enhanced ester conversion, improved biodiesel yield, suitable fuel properties, reduced production time/cost and reaction timing, optimum reaction conditions, etc. [13,14,15,16,17,18]. A direct transesterification method applied for microalgae and pongamia pinnata with combination of acidic and basic catalysts of boron triflouride and sodium methoxide, respectively, was found to be more effective than each individually used [19,20]. 

Improved fuel properties improve the performance and reduce the emissions of diesel engines. Generally, the fuel properties of biodiesel are not on par with diesel. These fuel properties may be suitably enhanced by adding some fuel additives. These include micron-sized particles, which act as catalysts and augment the combustion. However, these micron-sized particles may lead to agglomeration when they are used in liquid fuels. Further, they may form clusters at the bottom of the fuel, producing a non-uniform dispersion through the liquid fuel [21,22].

The size of a particle, ranging from 1 to 100 nm, is known as a nanoparticle. The prospects, challenges and advances of nano-based catalysts and their effect on engine stability, performance and emissions have been reviewed recently by Jabbar et al. and Manzoor et al. [23,24]. Prior research has focused mainly on two-step esterification-transesterification process for biodiesel production and then improving the fuel properties with nano additives. The present work focuses on production and characterization of biodiesel from crude oils of Neem and Karanja by a single-step direct transesterification process and further comparing the fuel properties of Neem and Karanja biodiesel-diesel blends. The effects of addition of nano particles (NPs) on fuel properties in different proportions in low concentrated B20 (20% biodiesel plus 80% diesel by volume) biodiesel-diesel blend are studied. Finally, performance and emission tests were performed for B20 Biodiesel-Diesel-Nano blends.

## 2. Materials and Methods 

The crude oils of Neem and Karanja were purchased from Parker biotech private limited, Chennai, India. Neem oil and Karanja oil are non-edible oils and, hence, preferred for biodiesel production to avoid the fuel or food conflict. Furthermore, the large-scale availability of these trees makes them cheaper for the biodiesel production. Both these trees grow in many parts of India and abroad. The oil content in Neem seeds is around 30%, whereas in Karanja seeds, it is from 35.8 to 44% [12]. Chemicals required for the chemical reactions such as methanol, sodium methoxide (0.1 N), boron triflouride (BF3), petroleum ether 60–80 °C (spectroscopic grade), anhydrous sodium sulphate, etc., were purchased from the local markets (Gagani Chemicals, Hubli, India. All chemicals used were analytically graded. 

### 2.1. Biodiesel Production by Direct Transesterification (DT)

Two-step transesterification reaction is commonly used to prepare biodiesel. Higher reaction timing and lower yield are the major problems associated with this method. Furthermore, there are problems associated with the separation of glycerin from biodiesel. These problems could be minimized to a large extent by employing direct transesterification, which uses acid and base catalysts simultaneously. 

Initially methanol and sodium methoxide (base catalyst) were mixed and taken in reactor. The mixture was shaken well for proper mixing. Later boron triflouride (BF3), which acts as an acid catalyst, was added to the reactor containing methanol and sodium methoxide mixture. Finally, crude oil was added to the reaction mixture. The crude oil, methanol, sodium methoxide and boron triflouride were taken in the ratio 1:4:0.1:0.1, respectively. The reaction was carried out for just two hours at 50 °C. After 2 hours of direct transesterification, the reaction was stopped. The author used petroleum ether to separate biodiesel from the reaction mixture in his previous published research. The method described was not economical and was consuming more time when performing evaporation and condensation of petroleum ether [20]. Therefore, in the current research, it was decided to avoid usage of petroleum ether. After 2 hours of direct transesterification reaction, the reaction mixture was washed with distilled water without petroleum ether. The biodiesel was separated from water. Sufficient quantity of sodium sulphate was added to remove the moisture contents. The mixture was filtered. Finally, biodiesel was obtained. This biodiesel was blended with different concentrations of diesel on volume basis, which were denoted as B0 (Diesel), B10 (10% biodiesel and 90% diesel blend), B20 (20% biodiesel and 80% diesel blend), B40 (40% biodiesel and 60% diesel blend), B60 (60% biodiesel and 40% diesel blend), B80 (80% biodiesel and 20% diesel blend) and B100 (pure biodiesel). 

### 2.2. The Nano-Additive: Graphene Oxide

In this study, Graphene oxide (GO) has been used as a fuel additive synthesized using chemical vapor deposition (CVD). GO possess excellent thermal and electrical conductivities, higher magnetism besides high surface area to volume ratio. It improves the convective heat transfer coefficient, calorific value, cetane number of fuel if it is blended with the fuel. Furthermore, it reduces ignition delay and enhances micro-explosion. 

The size of GO NPs is 23–40 nm, Young’s modulus 1.0 TPa, an inherent mobility of 200,000 cm^2^ v^−1^ s^−1^, surface area 2630 m^2^g^−1^, thermal conductivity 3300 Wm^−1^K^−1^ and purity of 95–98.5%

### 2.3. Equipment List and Properties for Analysis

The physico-chemical properties of Neem and Karanja and their respective methyl esters along with their blends have been found in accordance with the ASTM D6751 standards. Table 1 shows the list of equipment used.

### 2.4. Engine Testing for Performance and Emissions

**Figure 1:**
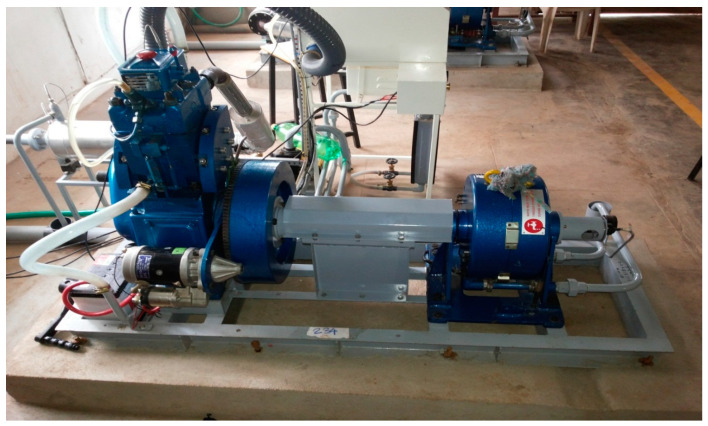
Variable compression ratio diesel engine.

Experiments were conducted on single cylinder four stroke variable compression ratio engine with electric starter connected to eddy current type dynamometer for loading as shown in Figure 1. The specification of instrument and equipment of the engine are given in Table 2. The engine was run at a fixed compression ratio of 17.5 and at a rated speed of 1500 rpm for variable loading conditions.

Throughout the experiments, the compression ratio, fuel injection pressure and ignition timing were kept constant at 17.5: 1, 205 bar and 23° before top dead center, respectively. To record different emissions such as HC and CO, an AVL DiGas444G gas analyzer (AVL India Private Limited—Bangalore, India) was used, which works on a non-dispersive infra-red technology (NDIR). Smoke in the exhaust gas was measured by the Smoke Meter AVL (AVL India Private Limited—Bangalore, India). All the readings were noted after the engine is reached steady state. The engine was loaded with the help of an eddy current dynamometer. 

#### Uncertainty Analysis 

The uncertainties and accuracies of the measured parameters in the current investigations are given in Table 3. The calculation for uncertainty for brake-specific fuel consumption is based on the systematic set of procedures available in open literature [25]. 

The variance of the measured factors estimated utilizing Gaussian distribution as described in Equation (1), under the of limits confidence of ±2σ. The limit 2σ reflects the average bound of 95% of the calculated values.

(1) $$\Delta X_{in}=\;\frac{2\sigma_{sd}}{\bar{X_{n}}}*100.\;$$

where $X_{n}$ is number of readings, $\bar{\Delta X_{in}}$ indicates trial readings and $\sigma_{sd}$ implies the standard deviation (SD). Uncertainties of determined factors were calculated utilizing the formula demonstrated in Equation (2):

*R* = *f*(*X*_1_, *X*_2_, *X*_3_,…………*X_n_*) (2)

(3) $$\Delta R=\sqrt{\left[{\left(\frac{\partial R}{\partial X_{1}}\Delta X_{1}\right)}^{2}+{\left(\frac{\partial R}{\partial X_{2}}\Delta X_{2}\right)}^{2}+{\left(\frac{\partial R}{\partial X_{3}}\Delta X_{3}\right)}^{2}+....................{\left(\frac{\partial R}{\partial X_{n}}\Delta X_{n}\right)}^{2}\right]}$$

where *R* in Equation (3) reveals the function of *X*_1_, *X*_2_,……*X_n_*, and *X*_1_, *X*_2_,……*X_n_* indicates the no. of obtained readings. Therefore, ΔR is determined by RMS (root mean square) of errors related to computed variables. The overall uncertainty of the present experimentation was found to be ±0.34%, and it is much smaller than the std. ± 5%. Hence, the overall uncertainty of the present research was within the acceptable limits [26].

By utilizing Equation (4), the uncertainties in various quantified and specific variables were analyzed. The errors bars were by averaging 3 readings and included in all the engine characteristics using NOME, KOME and graphene oxide fuel blends.

(4) $$\mathrm{Uncertainty}\;=\;\sqrt{\begin{array}{c} \mathrm{Uncertainty}\;\left(\%\right)\;\mathrm{of}\;(\mathrm{sq}.\mathrm{BTE}+\mathrm{sq}.\mathrm{BSFC}\\ +\mathrm{sq}.\;\mathrm{CO}+\mathrm{sq}.\mathrm{HC}+\mathrm{sq}.\mathrm{smoke}) \end{array}}\;=\;\sqrt{\begin{array}{c} \mathrm{Uncertainty}\;\left(\%\right)\;\mathrm{of}\;(\mathrm{sq}.0.20+\mathrm{sq}.0.15\\ +\mathrm{sq}.\;0.15+\mathrm{sq}.0.12+\mathrm{sq}.0.15) \end{array}}\;=\;\pm \;0.34\%$$

## 3. Results and Discussion

### 3.1. Characterization of NOME and KOME

Table 4 and Table 5 give the characterization of Neem and Karanja biodiesel with their respective blends with diesel. 

**Figure 2:**
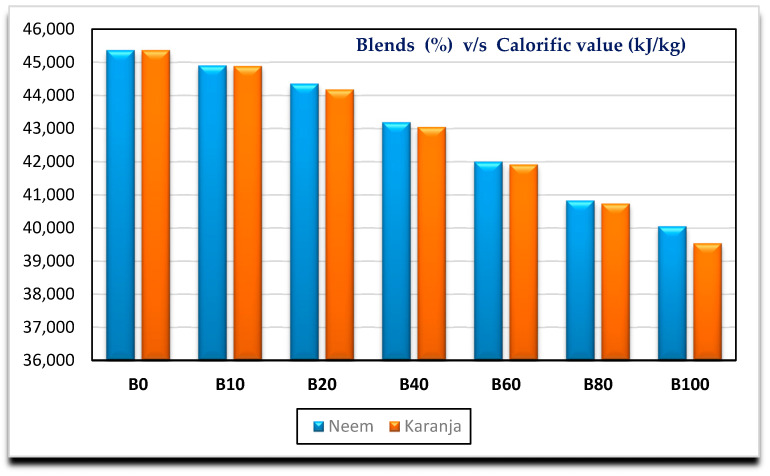
Variation of calorific value with biodiesel-diesel blends.

**Figure 3:**
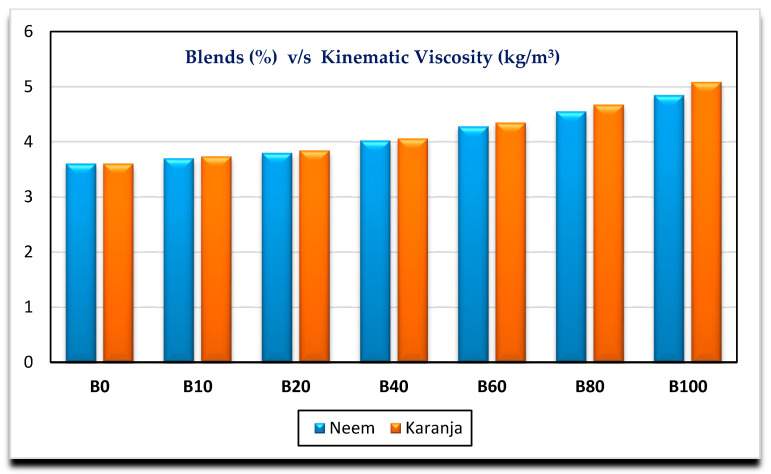
Variation of kinematic viscosity with biodiesel-diesel blends.

**Figure 4:**
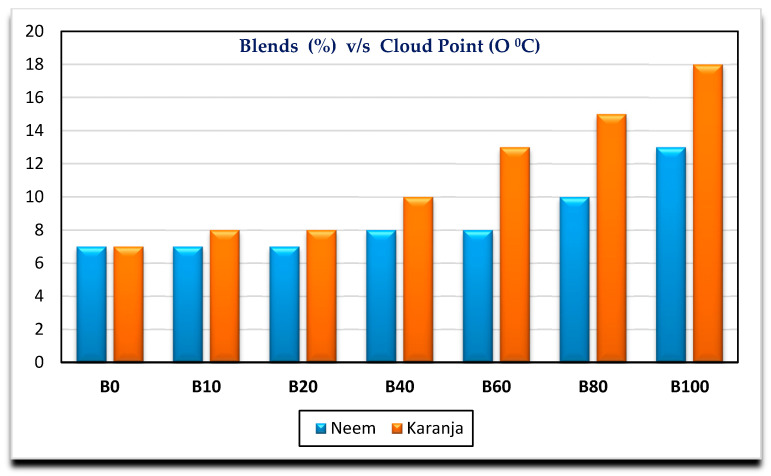
Variation of cloud point with biodiesel-diesel blends.

**Figure 5:**
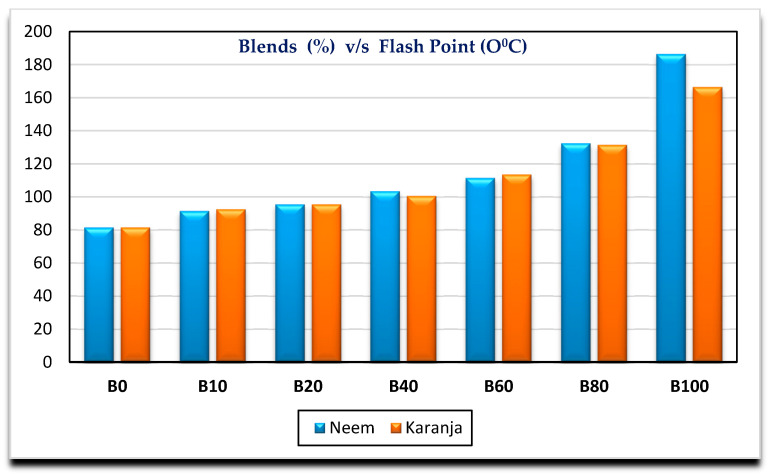
Variation of flash point with biodiesel-diesel blends.

Figure 2, Figure 3, Figure 4 and Figure 5 show the variation of calorific value, kinematic viscosity, cloud point and flash point with variation of biodiesel-diesel blends, respectively. 

The calorific values of diesel, Neem biodiesel and Karanja biodiesel are 45,369, 40,050 and 39,538 kJ/kg, respectively, and are shown in Figure 2. The calorific values of biodiesel are reduced by 13 to 15% compared to the calorific value of diesel due to the presence of oxygen contents [27]. Higher viscosity of fuel needs higher injection pressure for spray atomization of fuel. Higher viscosity affects its fluidity especially at low operating temperatures [28,29]. Biodiesel possesses higher viscosity than diesel. Kinematic viscosity of Neem (4.8489 mm^2^/s) biodiesel is less by 4.97% compared to Karanja (5.0901 mm^2^/s) biodiesel at 40 °C as shown in Figure 3. Both results are well within the limit of ASTM standards. Cold flow properties such as cloud point, pour point and cold filter plugging point are very important fuel properties for operating the engine at lower temperatures. At lower temperatures, the fuel may become partially solidified, resulting in the blockage of fuel supply to the engine, which in turn, may damage the engine fuel supply system [30]. Figure 4 shows the variation of cloud point with variation in the biodiesel-diesel blends. It is evident that Neem biodiesel possess excellent cloud point compared to Karanja biodiesel especially for higher blends. The CP of Karanja biodiesel-diesel blends are more by 25, 62, 50 and 38 for B40, B60, B80 and B100 when compared to the respective Neem biodiesel-diesel blends. However, there was no significant difference for low blends. The flash point is another important fuel property. It is the minimum temperature at which the fuel possesses sufficient vapors for its combustion. It is also important for fuel handling issues. The lower blends of biodiesel show no significant difference in flash point temperature as shown in Figure 5. However, Karanja biodiesel is slightly better compared to Neem biodiesel.

### 3.2. Effects of GO Nano on Fuel Properties 

The nano-additives are used to improve some of the biodiesel fuel properties, which further improve the performance of the engine besides reduced emissions with no or little engine modifications. Nano particles (NPs), which possess enhanced surface area/volume ratio, also act as a catalyst. The higher surface area/volume ratio improves the quality of air and fuel mixture and, hence, increases the rate of chemical reaction during combustion [31]. Higher oxygen contents make the combustion complete, which is very important for minimizing the formation of carbon monoxide. The blending of biodiesel-diesel blends, and nano particles are very significant, as they should be free from any agglomeration.

In the current investigation, an ultrasonication technique is used to stably blend the nanoparticles in the fuel blends. Initially, different concentrations of nanofluids are prepared using bath and probe sonication method. The nanofluids are blended with a diesel–biodiesel (B20) blend, initially the nanofluids (35, 75 and 105 ppm) are transferred to diesel–B20 fuel blend, to remove excess moisture, the blends are heated at 60 °C using magnetic stirrer; later, it is sonicated for one hour using bath sonication and, finally, the nano fuel blends are stably dispersed using probe sonication at a frequency of 15–20 Hz for 20 min for each fuel blend. These steps enable stable dispersion of nanoparticles in fuel blends, thus preventing the nanoparticles from agglomerating. 

Ultrasonication technique avoids the agglomeration to the larger extent and, hence, is used in the present research for the preparation of NPs dispersed in base biodiesel fuel. Metal based nano additives results in agglomeration, clustering and settling due to their short-term stability. Hence, in the present research, the author has used graphene oxide, a carbon-based NP, to make the Biodiesel-Diesel-Nano blends. Nano additives are blended with a B20 biodiesel-diesel blend in the three different dosage levels of 35, 70 and 105 ppm. They are denoted as NOME20GO35, NOME20GO70 and NOME20GO105 for Neem Biodiesel-Diesel-Nano blends. Similarly, KOME20GO35, KOME20GO70 and KOME20GO105 are for Karanja biodiesel-diesel–graphene oxide nano blends.

Table 6 and Table 7 show the characterization of Neem and Karanja Biodiesel-Diesel-Nano blends, respectively. 

**Figure 6:**
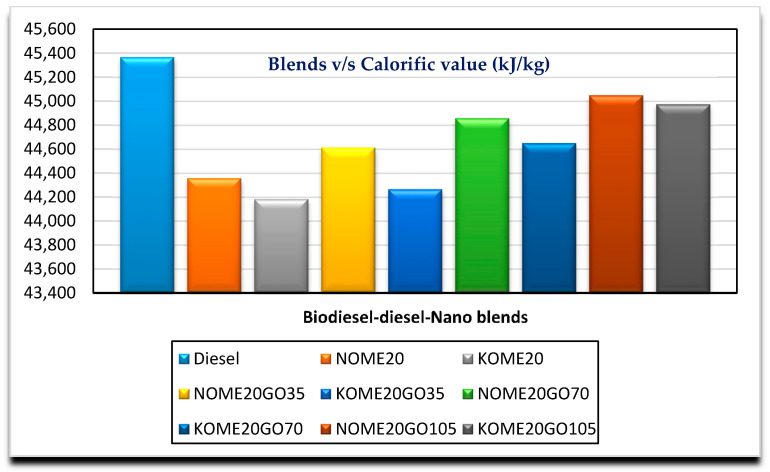
Variation of calorific value with Biodiesel-Diesel-Nano blends.

Figure 6 shows the effect of nano addition to the biodiesel-diesel blend (B20) in different proportions. The calorific value of the diesel is always more than other biodiesel-diesel blends. However, the calorific value of biodiesel-diesel blends increase with addition of nano additives. Addition of NPs improves the ignition quality, coefficient of thermal conductivity and energy density of resulting mixture. Hence, an increased calorific value was obtained for different Biodiesel-Diesel-Nano blended fuel mixture [32]. NOME20 nano blended fuel is having slightly higher calorific value when compared with KOME20 nano blended fuel. It could be further stated that the higher blends of GO NPs NOME20GO105 (45,050 kJ/kg) and KOME20GO105 (44,975 kJ/kg) have the calorific values at par with neat diesel. These results were in agreement with the results obtained by Kannan et al. [33].

**Figure 7:**
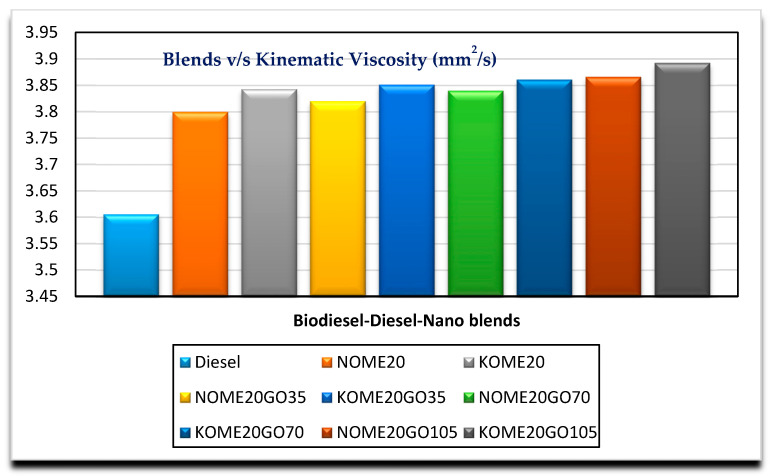
Variation of kinematic viscosity with Biodiesel-Diesel-Nano blends.

Higher kinematic viscosity of the fuel affects the fuel supply system in the engine. The fuel injection system and charge preparation are reliant on density, volatility and viscosity of fuel, which are often interdependent. The kinematic viscosity of biodiesel-diesel blends increase with the addition of nano additives as shown in Figure 7. It is apparent that the NPs added to the biodiesel-diesel blends increase the fluid resistance and, hence, increase the kinematic viscosity [31]. The kinematic viscosity of NOME20 is marginally better compared to KOME20 nano blends. An increase in concentration of NPs in biodiesel-diesel blends increases the kinematic viscosity [34,35]. 

**Figure 8:**
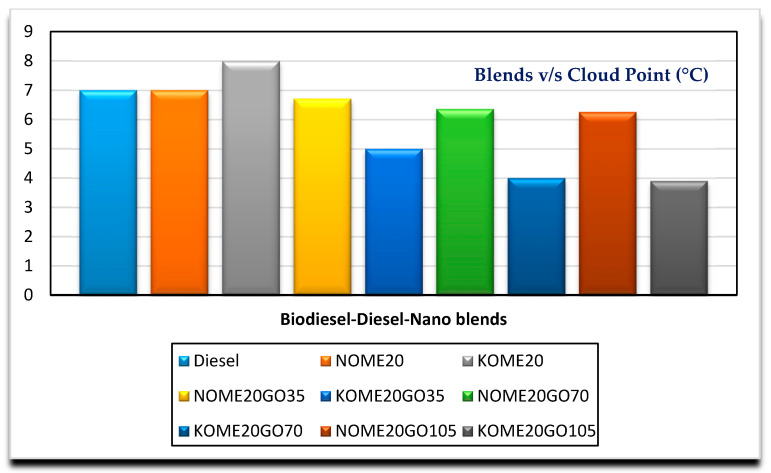
Variation of cloud point with Biodiesel-Diesel-Nano blends.

**Figure 9:**
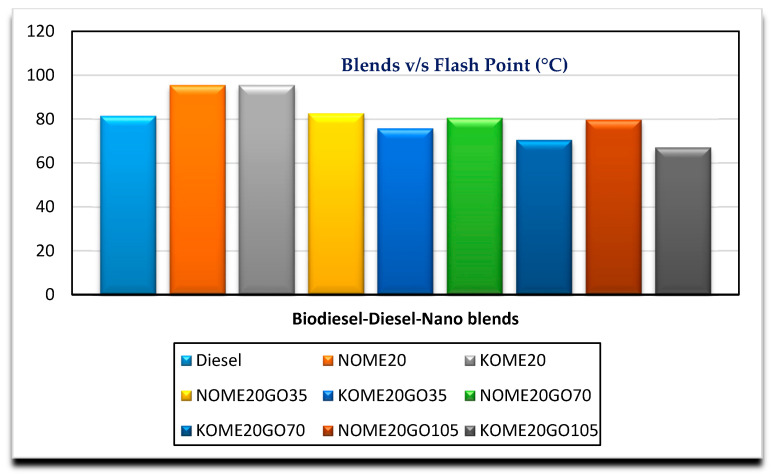
Variation of flash point with Biodiesel-Diesel-Nano blends.

Figure 8 and Figure 9 show the variation of cloud point and flash point with varying nano blends, respectively, for both NOME20 and KOME20. It can be observed that cloud point and flash point decreased marginally with nano addition. Cloud point for NOME20 reduced from 1.5 to 5.2% with the addition of NPs at different concentrations, whereas for KOME20, cloud point reduced by 37% by the addition of 35 ppm NPs. Further, it reduced by 20 and 2.5% for the addition of 70 and 105 ppm of NPs, respectively. The flash point of for both NOME20 and KOME20 is 95.5 °C, which reduced by 13.4 and 20.6% by the addition of 35 ppm of NPs. There was no significant change in flash point for higher concentrated NPs fuel blends. Similar trends were reported in the studies conducted by Kesin et al. and Guru et al. [36,37]. However, there were reports where there was no significant change in cloud point by the addition of NPs to the biodiesel-diesel blends, and flash point was found to increase with addition of dosage of NPs [31]. This may be attributed to the type of feedstocks and NPs used. The higher concentrated nano blended NOME20 and KOME20 are better compared to diesel in terms of cloud point and flash point. 

#### 3.2.1. Brake Thermal Efficiency

**Figure 10:**
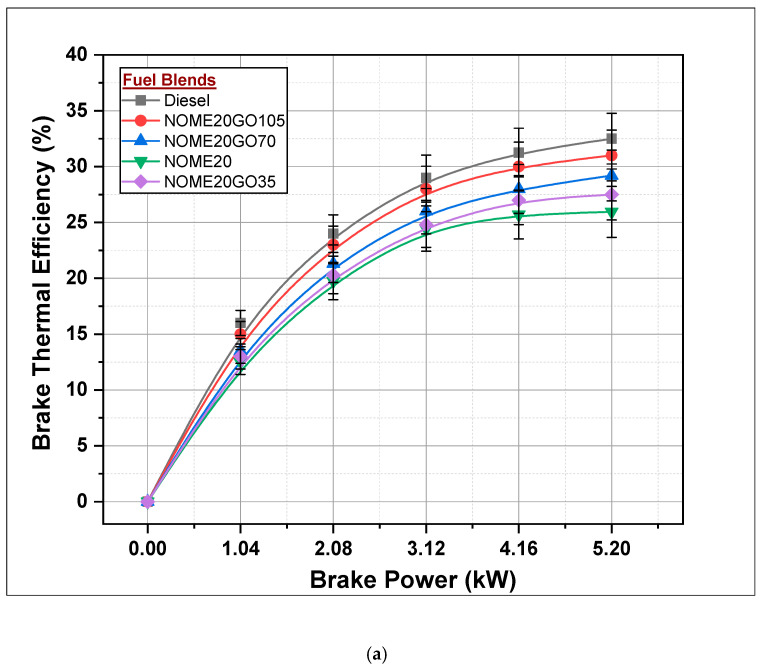

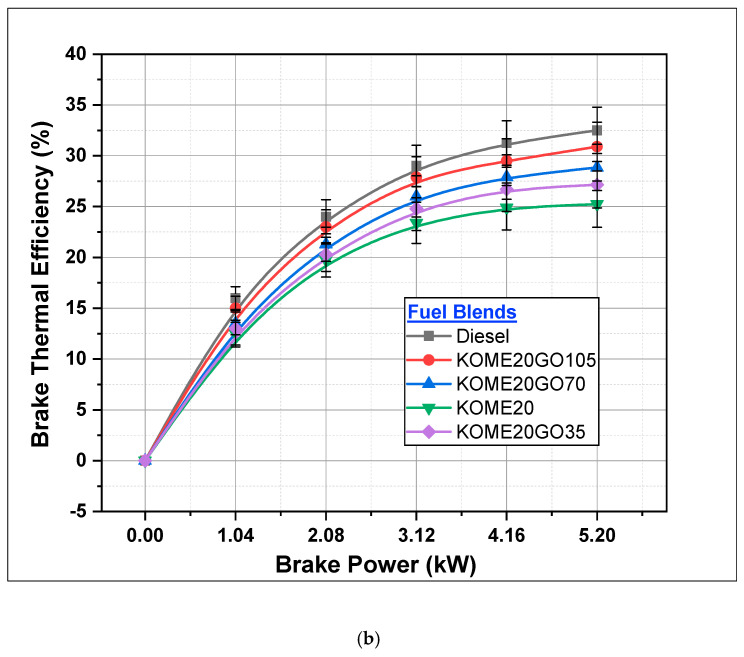
Variation of brake thermal efficiency with Biodiesel-Diesel-Nano blends for (**a**) NOME20 (**b**) KOME20.

The variation of brake thermal efficiency (BTE) with load for diesel, NOME20, KOME20, along with different dosage levels (35, 70 and 105 ppm) of graphene oxide NPs has shown in Figure 10a,b respectively. It is observed that BTE of the engine increases with the increase in engine brake power (BP). Brake thermal efficiencies for diesel, NOME20 and KOME20 are 32.5, 25.95 and 25.24%, respectively, at full-load conditions. Further, it has been observed that BTE of NOME20 and KOME20 increases with addition of NPs. Brake thermal efficiencies of NOME20GO105 and KOME20GO105 are found to be 31 and 30.9%, respectively, and reduced by 4.8 and 5.1% than the BTE of diesel run engine. Furthermore, it can be observed that the BTE of NOME20 and KOME20 increased by 19.5 and 22.6% by the addition of 105 ppm of NPs. A similar trend was reported by Al-Seesy et al. in their work [38]. The concentration of NPs improves the combustion characteristics of fuel (Biodiesel-Diesel-Nano blends) [39]. The reduced ignition delay, fast burning process and complete combustion are the reasons that could be attributed to improved BTE. 

#### 3.2.2. Brake-Specific Fuel Consumption

**Figure 11:**
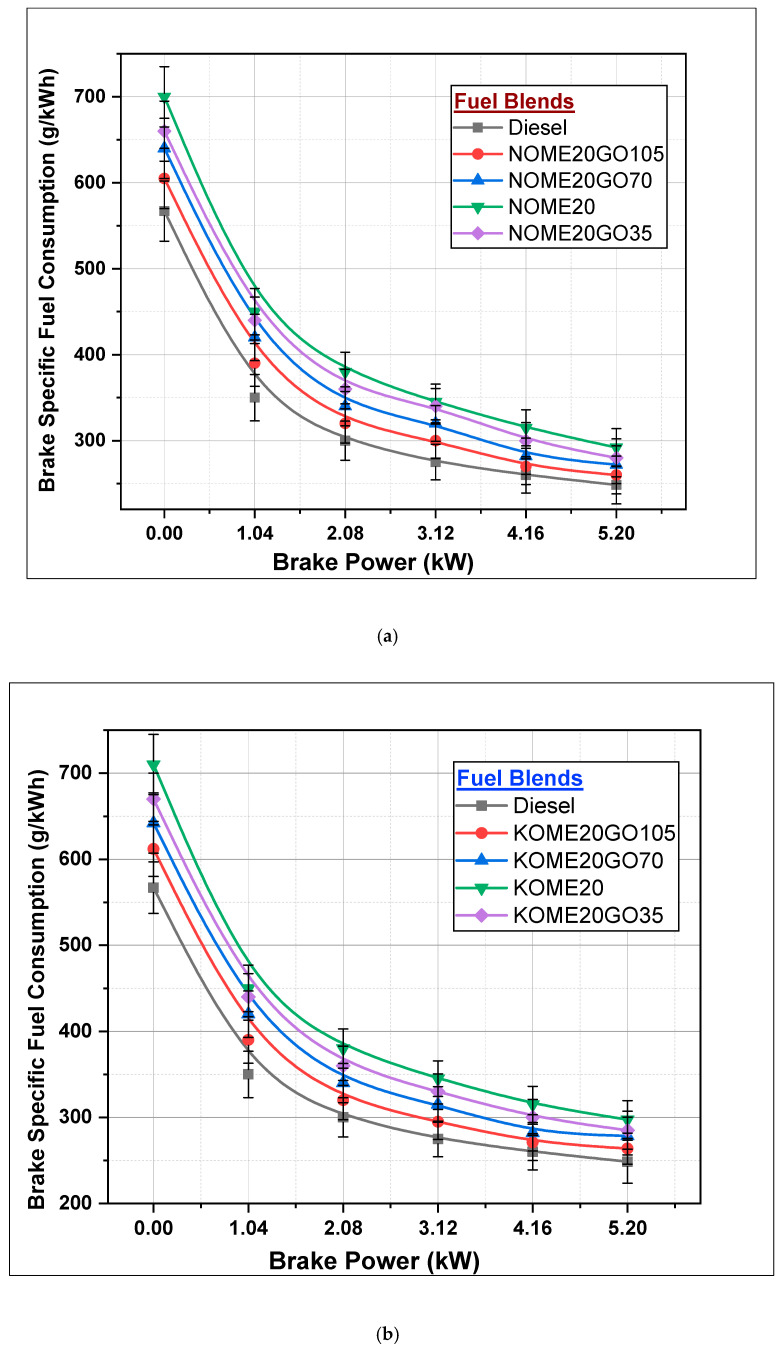
Variation of brake-specific fuel consumption with Biodiesel-Diesel-Nano blends (**a**) NOME20 (**b**) KOME20.

The brake-specific fuel consumption (BSFC) is an important performance parameter of an IC engine. Figure 11 shows the variation of BSFC with variation in BP. The BSFC is less in diesel-fueled engine (248.5 g/kWh) at maximum engine load. The BSFC for NOME20, NOME20GO35, NOME20GO70 and NOME20GO105 are 292.5, 280.5, 272.5 and 260.5 g/kWh, respectively shown in Figure 11a. Similarly, the BSFC for KOME20, KOME20GO35, KOME20GO70 and KOME20GO105 are 297.5, 285, 278.44 and 263.78 g/kWh, respectively shown in Figure 11b. It can be noted that neem Biodiesel-Diesel-Nano blends are slightly better (BSFC less by 1.25% to 2.17%) as compared to Karanja Biodiesel-Diesel-Nano blends. The BSFC is more in case of biodiesel-diesel blends (NOME20 and KOME20) by 17 to 19.71% compared to diesel owing to their higher viscosity and poor atomization, and hence, more fuel is consumed during diffusion stage of combustion. However, the addition of NPs in biodiesel-diesel blends reduce the fuel consumptions. The NPs promote the oxygen for biodiesel-diesel blends resulting in higher power output with reduced fuel consumptions [40]. 

#### 3.2.3. Hydrocarbons (HC)

**Figure 12:**
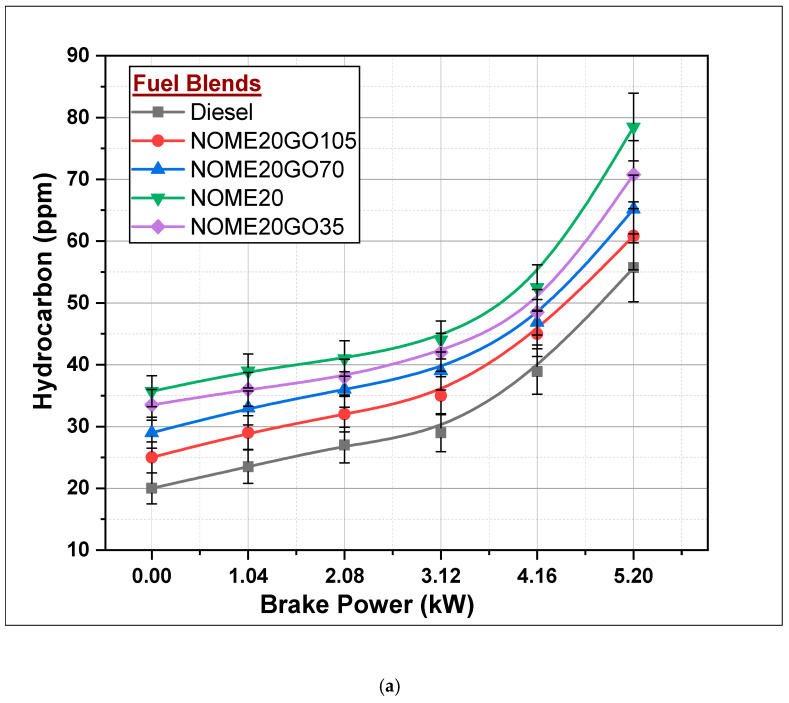

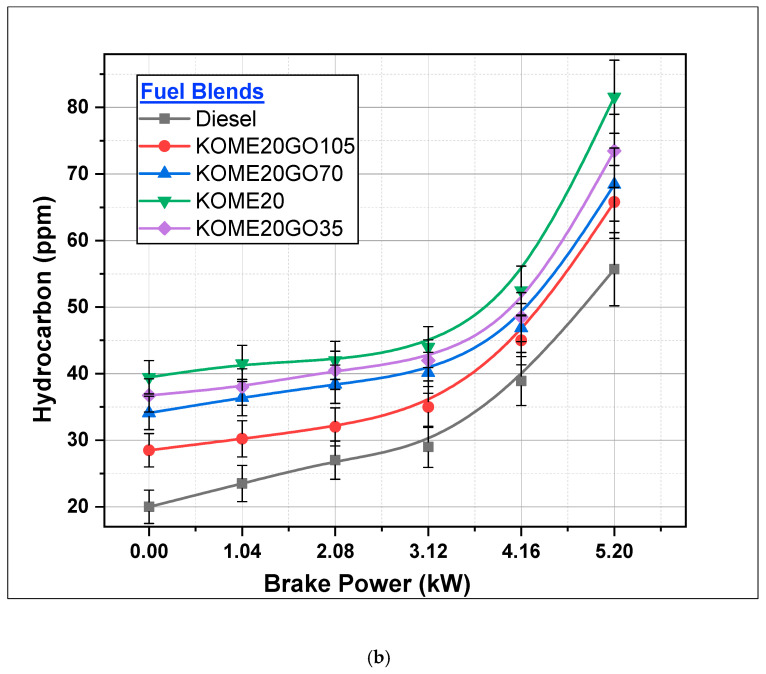
Variations in hydrocarbon formation with Biodiesel-Diesel-Nano blends (**a**) NOME20 (**b**) KOME20.

Hydrocarbons are the result of incomplete combustion of the fuel. Figure 12a,b represent the formation of HC for diesel and different Biodiesel-Diesel-Nano blends at different engine loads for NOME20 and KOME20 respectively. For all engine loads, the diesel-fueled engine performed better compared to other fuel blends. NOME20 and KOME20 biodiesel-diesel blends resulted 78.47 and 81.6 ppm, respectively, at maximum engine load, NOME20 found slightly better compared to KOME20. The formation of HC was reduced by 28.9 and 24% for NOME20 and KOME20 higher concentrated NPs, respectively, at full engine load. NOME20GO135 and KOME20GO135 led to 60.84 and 65.8 ppm of HC formation and maximum engine load. NOME20GO135 resulted in 4.96 ppm less HC compared to KOME20GO135 at maximum engine load. The reduction in HC may be attributed to proper mixing of fuel blends with air and increased surface/volume ratio of NPs [41,42,43]. 

#### 3.2.4. Smoke Formation

**Figure 13:**
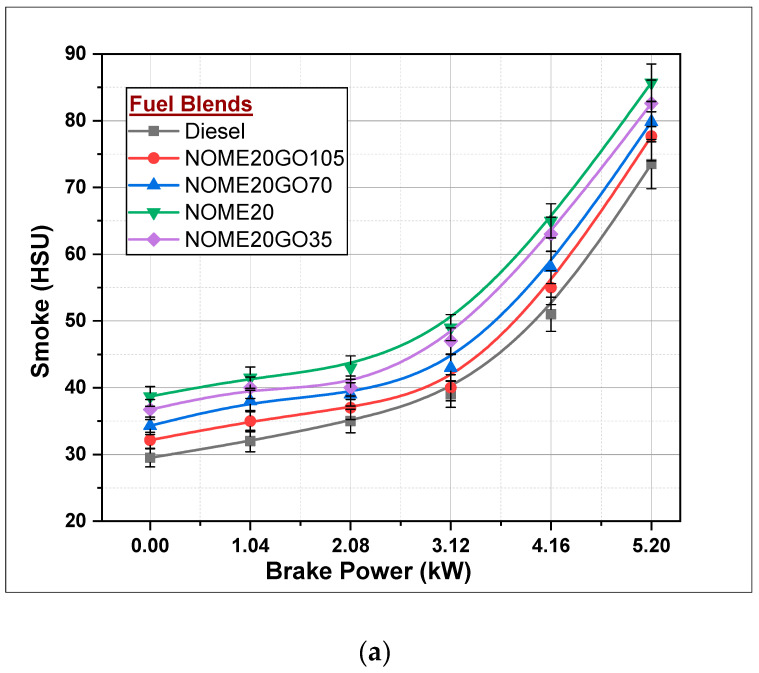

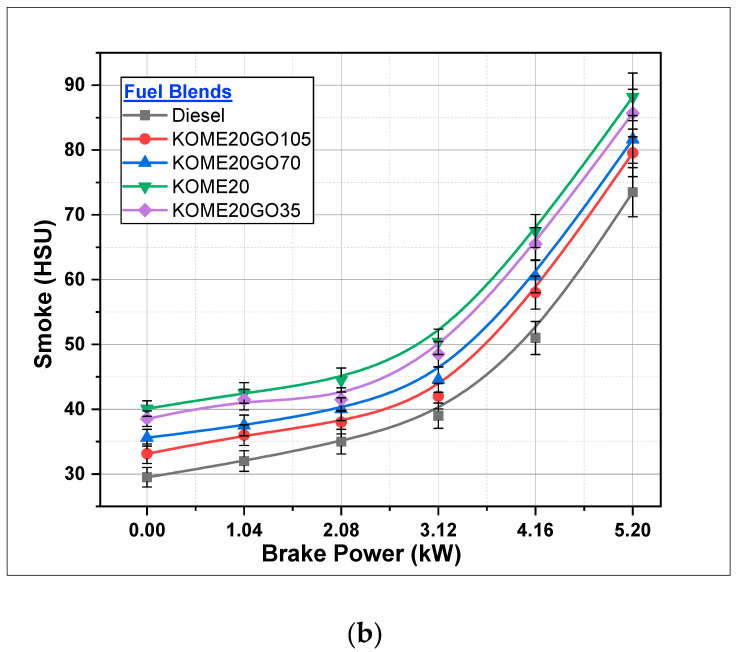
Variations in smoke formation with Biodiesel-Diesel-Nano blends (**a**) NOME20 (**b**) KOME20.

Diesel engines are the major source of formation of smoke and are more spontaneous at maximum engine load conditions, which can be observed from Figure 13. Formation of smoke is less at idling and lower load conditions and spontaneously increases at full-load conditions. At full-load conditions, smoke for NOME20 and KOME20 are 85.7HSU and 88.2HSU, respectively. The reasons for higher smoke in biodiesel blended diesel engine could be higher viscosity and density, low volatility, which results in poor atomization of fuel and, hence, may result is loss of power output and so on. However, NPs act as oxygen donors for biodiesel-diesel blends, thus improving the fuel properties. Addition of 105 ppm of NPs to NOME20 and KOME20 reduces the smoke from 88.2HSU to 77.67HSU for NOME20, and similarly for KOME20, it reduces from 88.2HSU to 79.56HSU. This has been shown in Figure 13a,b for NOME20 and KOME20 respectively. Graphene oxide NPs contain higher oxygen contents that reduce the ignition delay during combustion, which helps in completing the combustion of fuels [44,45]. A maximum of 73.5HSU of smoke was recorded for diesel at full engine condition.

#### 3.2.5. Carbon Monoxide (CO) Formation

**Figure 14:**
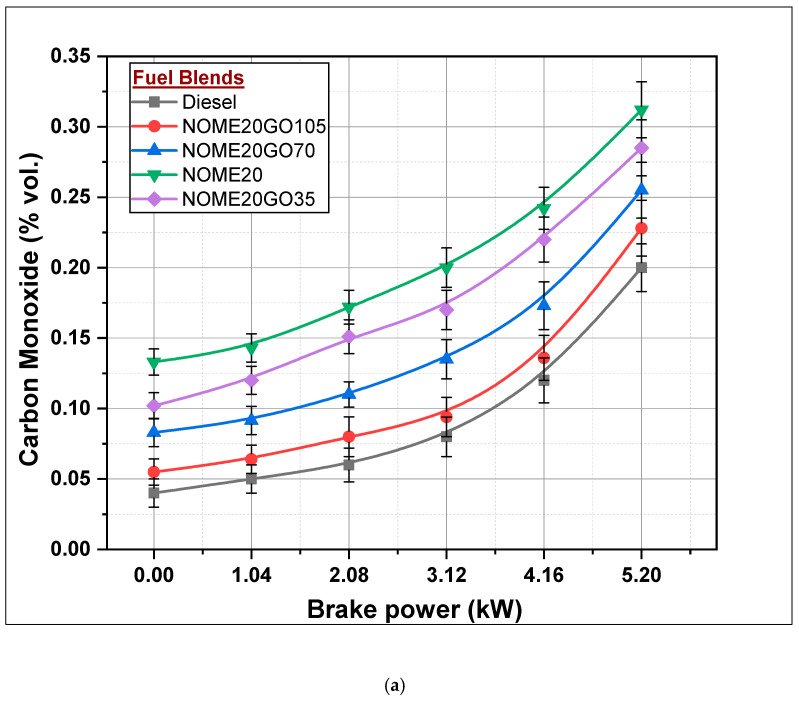

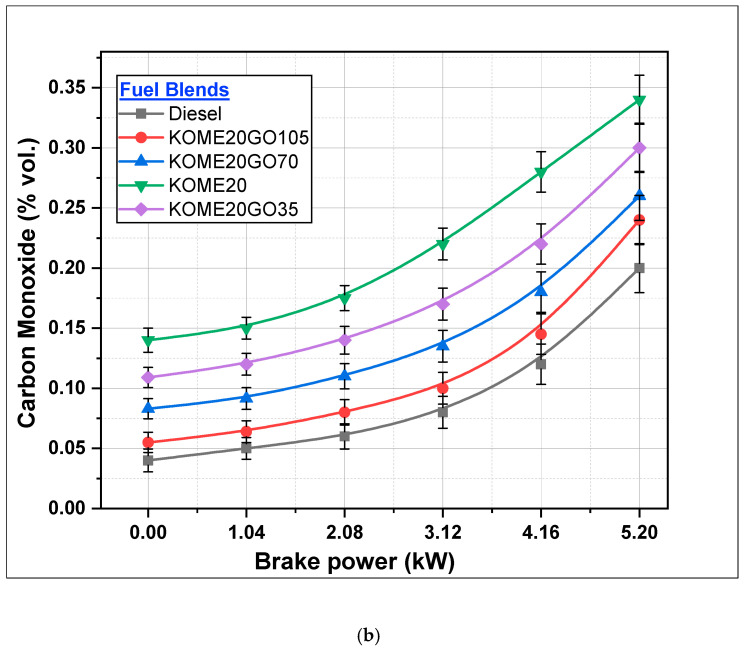
Formation of carbon monoxide (CO) with Biodiesel-Diesel-Nano blends (**a**) NOME20 (**b**) KOME20.

Unavailability of sufficient quantity of oxygen for combustion inside the combustion chamber is the main reason for incomplete combustion and, hence, formation of CO. The formation of CO with brake power for different fuel blends has been shown in Figure 14. Formation of CO increases with increase in BP for all combinations of fuels and diesel. The complex oxidation reaction and higher viscosity of biodiesel-diesel blends result in more quantity of CO formation. In case of NOME20, 0.312% CO was formed, whereas in the case of KOME20, it was found to be 0.34% which have been shown in Figure 14a,b respectively. Subsequent addition of NPs to biodiesel-diesel blends reduced the formation of CO. A percentage of 0.228 and 0.24% of CO was formed in NOME20GO105 and KOME20GO105, respectively. The addition of NPs enhances combustion quality of the fuel, hence there was a reduction in CO formation in Biodiesel-Diesel-Nano blends. 

## 4. Conclusions

The current research is a comparison of fuel properties biodiesel and their blends prepared by using the non-edible feedstocks of Neem and Karanja by employing direct transesterification process. Furthermore, B20 biodiesel-diesel blends were blended with graphene oxide NPs in different proportions. The following points are drawn from the results.

- Biodiesel prepared by direct transesterification reduced the reaction timing.
- Neem biodiesel is slightly better compared to Karanja in terms of calorific value, kinematic viscosity. There is a marginal difference in cloud point and flash point.
- The fuel properties of both blends improved with addition of higher dosages of graphene oxide nano particles.
- Maximum BTE of diesel, NOME20GO105 and KOMEGO105 is 32.5, 31 and 30.9%, respectively, at full load.
- BSFC for NOME20GO105 and KOME20GO105 was reduced by 10 and 11%, respectively, compared to NOME20 and KOME20.
- CO for NOME20GO105 and KOME20GO105 was reduced by 27 and 29%, respectively, compared to NOME20 and KOME20.
- BTE, BSFC, CO, HC and smoke were slightly better for the diesel-fueled engine compared to NOME20GO105 and KOME20GO10 run engines.

## Figures and Tables

**Table 1:** Measuring instruments list.

No	Property	Equipment	Manufacturer
1	Calorific value	C2000 basic calorimeter—automatic	(IKA, UK)
2	Kinematic viscosity	SVM 3000 automatic	(Anton Paar, London, UK)
3	Density	SVM 3000—automatic	(Anton Paar, London, UK)
4	Cloud and Pour point	Cloud and Pour point tester—automaticNTE 450	(Normalab, Valliquerville, France)
5	Cold Filter Plug in Point	Cold filter plugging point—automatic NTL 450	(Normalab, Valliquerville, France)
6	Flash Point	Pensky-martens flash point automatic NPM 440	(Normalab, Valliquerville, France)

**Table 2:** Technical specifications of the engine.

Type	Single Cylinder, Four Stroke, Variable Compression Ratio Diesel Engine
Bore	87.5 mm
Stroke	110 mm
Compression ratio	17.5:1
Maximum power	5.2 kW
Maximum speed	1500 rpm
Injection system	Direct injection
Start of injection	23° before TDC
Injection pressure	205 bar
Cooling system	Water cooled

**Table 3:** Uncertainty and accuracy levels of calculated engine parameters.

Parameters	Accuracy (±)	Uncertainty (%)
CO emission (%)	±0.01%	±0.15
HC emission (ppm)	±10 ppm	±0.12
Smoke meter (HSU)	±1	±0.15
Brake thermal efficiency (%)	-	±0.20
Brake-specific fuel consumption (g/kW-h)	-	±0.15

**Table 4:** Fuel properties of Neem biodiesel-diesel blends by DT method.

	B0	B10	B20	B40	B60	B80	B100
Calorific value (kJ/kg)	45,369	44,909	44,357	43,191	42,003	40,833	40,050
Kinematic viscosity (mm^2^/s) at 40 °C	3.6056	3.7028	3.7997	4.024	4.2807	4.556	4.8489
Density (kg/m^3^) at 15 °C	851.8	854.6	857.6	863.4	869.2	875.4	880.9
Specific gravity at 15 °C	0.8526	0.8554	0.8584	0.8642	0.8700	0.8762	0.8817
Cloud point (°C)	7	7	7	8	8	10	13
Pour point (°C)	2	2	5	8	8	11	14
CFPP (°C)	0	5	4	4	5	8	12
Flash point (°C)	81.5	91.5	95.5	103.5	111.5	132.5	186.5

**Table 5:** Fuel properties of Karanja biodiesel-diesel blends by DT method.

	B0	B10	B20	B40	B60	B80	B100
Calorific value (kJ/kg)	45,369	44,888	44,183	43,052	41,920	40,737	39,538
Kinematic viscosity (mm^2^/s) at 40 °C	3.6056	3.7380	3.8430	4.0615	4.3480	4.6745	5.0901
Density (kg/m^3^) at 15 °C	851.8	855.6	859.6	867.4	876.0	884.10	891.7
Specific gravity at 15 °C	0.8526	0.8564	0.8604	0.8682	0.8768	0.8849	0.8935
Cloud point (°C)	7	8	8	10	13	15	18
Pour point (°C)	2	5	5	8	8	8	11
CFPP (°C)	0	6	8	10	12	13	16
Flash point (°C)	81.5	92.5	95.5	100.5	113.5	131.5	166.5

**Table 6:** Fuel properties of Neem Biodiesel-Diesel-Nano blends by DT method.

	ASTM D6751	NOME20	NOME20 GO35	NOME20 GO70	NOME20 GO105	ASTM Test Limit
Calorific value (kJ/kg)	D5865	44,357	44,614	44,858	45,050	Min. 35,000
Kinematic viscosity (mm^2^/s) at 40 °C	D445	3.7997	3.82	3.84	3.866	1.9–6
Density (kg/m^3^) at 15 °C	D4052	857.6	858.12	861.44	868.3	860–900
Specific gravity at 15 °C	D891	0.8584	0.867	0.88	0.894	0.87–0.90
Cloud point (°C)	D2500-11	7	6.71	6.636	6.26	−3 to 12
Pour point (°C)	D97-12	5	4.12	3.86	3.794	−15 to 16
CFPP (°C)	D6371	4	2.67	2.15	1.92	--
Flash point (°C)	D93	95.5	82.62	80.66	79.74	Min. 93

**Table 7:** Fuel properties of Karanja Biodiesel-Diesel-Nano blends by DT method.

	ASTM D6751	KOME20	KOME20 GO35	KOME20 GO70	KOME20 GO105	ASTM Test Limit
Calorific value (kJ/kg)	D5865	44,183	44,266	44,650	44,975	Min. 35,000
Kinematic viscosity (mm^2^/s) at 40 °C	D445	3.8430	3.8512	3.861	3.8924	1.9–6
Density (kg/m^3^) at 15 °C	D4052	856.6	858.11	858.38	859.0	860–900
Specific gravity at 15 °C	D891	0.8604	0.871	0.882	0.891	0.87–0.90
Cloud point (°C)	D2500-11	8	5	4	3.9	−3 to 12
Pour point (°C)	D97-12	5	3.89	4.15	4.61	−15 to 16
CFPP (°C)	D6371	8	6.8	6.1	6	--
Flash point (°C)	D93	95.5	75.8	70.5	67.1	Min. 93

## Future Recommendation

The enduring research on the lifecycle of the engine and its components are needed to determine such long-term adverse effects of nanoparticles under different operating conditions.

## Nomenclature

NOME	Neem Oil methyl ester
KOME	Karanja Oil methyl ester
DT	Direct transesterification
B0	Diesel
B100	Pure biodiesel
GO	Graphene oxide
NPs	Nano particles
NOME20	Neem biodiesel 20% blended with 80% diesel
KOME20	Karanja biodiesel 20% blended with 80% diesel
NOME20 GO35	NOME20 with 35 ppm of GO NPs
NOME20GO70	NOME20 with 70 ppm of GO NPs
NOME20GO105	NOME20 with 105 ppm of GO NPs
KOME20GO35	KOME20 with 35 ppm of GO NPs
KOME20GO70	KOME20 with 70 ppm of GO NPs
KOME20GO105	KOME20 with 105 ppm of GO NPs
